# p53-Dependent subcellular proteome localization following DNA damage

**DOI:** 10.1002/pmic.201000213

**Published:** 2010-11

**Authors:** François-Michel Boisvert, Angus I Lamond

**Affiliations:** Wellcome Trust Centre for Gene Regulation and Expression, College of Life Sciences, University of DundeeMSI/WTB/JBC Complex, Dundee, UK

**Keywords:** Cell biology, DNA damage, Protein localization, p53, Stable isotope labeling with amino acids in cell culture

## Abstract

The nucleolus is involved in regulating several aspects of stress responses and cell cycle arrest through the tumor suppressor p53. Under normal conditions, p53 is a short-lived protein that is present in cells at a barely detectable level. Upon exposure of cells to various forms of exogenous stress, such as DNA damage, there is a stabilization of p53 which is then responsible for an ensuing cascade of events. To further investigate the effect of p53 activation, we used a MS-based proteomics method to provide an unbiased, quantitative and high-throughput approach for measuring the subcellular distribution of the proteome that is dependent on p53. The spatial proteomics method analyses a whole cell extract created by recombining differentially labeled subcellular fractions derived from cells in which proteins have been mass labeled with heavy isotopes [Boisvert, F.-M., Lam, Y. W., Lamont, D., Lamond, A. I., *Mol. Cell. Proteomics* 2010, 9, 457–470]. This was used here to measure the relative distribution between cytoplasm, nucleus and nucleolus of around 2000 proteins in HCT116 cells that are either expressing wild-type p53 or null for p53. Spatial proteomics also facilitates a proteome-wide comparison of changes in protein localization in response to a wide range of physiological and experimental perturbations. We used this method to study differences in protein localization in HCT116 cells either with or without p53, and studied the differences in cellular response to DNA damage following treatment of HCT116 cells with etoposide in both p53 wild-type and null genetic backgrounds.

## 1 Introduction

The nucleolus is involved in regulating several aspects of stress responses and cell cycle arrest through the tumor suppressor p53. The *p53* tumor suppressor gene is mutated in approximately 50% of human tumors and plays an important role in the response to genotoxic stress and hypoxia 1. Under normal conditions, p53 is a short-lived protein that is present in cells at a barely detectable level. Upon exposure of cells to various forms of exogenous stress, such as DNA damage, heat shock, hypoxia, etc., there is a stabilization of p53 which is then responsible for an ensuing cascade of events, resulting in either cell cycle arrest or in apoptosis. Accumulation of p53 in the cell induces the p21-mediated inhibition of cyclin D/cdk4 and cyclinE/cdk2, resulting in cell cycle arrest in G1. The stability of the p53 protein in mammals is primarily regulated in non-transformed cells by the interplay of two proteins, hdm2 and p14Arf in humans (the equivalent mouse proteins are mdm2 and p19Arf) 2. Hdm2 functions as a specific E3 ubiquitin ligase for p53, resulting in a low level of p53 under normal growth conditions due to proteasome-mediated degradation of ubiquitin-conjugated p53. A variety of stimuli, including stress pathways and oncogenic signals, increase expression of Arf, which then associates with hdm2 to inhibit the ubiquitination, nuclear export and subsequent degradation of p53. It has been proposed that Arf physically sequesters hdm2 in nucleoli (No), thereby relieving nucleoplasmic p53 from hdm2-mediated degradation 3. Arf is predominantly a nucleolar protein and might also regulate ribosome biogenesis by retarding the processing of early 47S/45S and 32S rRNA precursors, perhaps through interaction with B23 4. Exposure of cells to various forms of stress, such as DNA damage, heat shock and aberrant ribosome biogenesis results in an increase in p53 and cell cycle arrest. Thus, the nucleolus acts as a sensor for cellular stress signals through p53 stabilization 5.

SILAC, or stable isotope labeling with amino acids in cell culture, is the use of stable isotopic atoms along with MS for quantitative MS analysis 6, 7. This method allows quantitative analyses of proteins by comparison of the mass of light and heavier forms of the same peptide from a given protein, arising from the presence of heavier, stable isotopes, such as ^13^C, ^2^H and ^15^N. These stable isotopes are incorporated in proteins by *in vivo* labeling, *i.e*. growing the cells in specialized media where specific amino acids, typically arginine and lysine, are replaced with corresponding heavy isotope-substituted forms in which either all carbons or combinations of carbons, hydrogens or nitrogens are isotope labeled 8. Cleavage at the substituted arginine or lysine by trypsin generates a peptide with a shift in mass relative to the control (*i.e*. unsubstituted) peptide and this can be easily resolved by MS. The ratio of intensities of the “light” and “heavy” peptide signals identified by MS directly correlates with the relative amount of the cognate protein from each sample. This method has been widely used for both relative quantification of protein levels after exposure of cells to drugs and inhibitors and for the identification of specific protein interaction partners 9–12.

Using a quantitative proteomic approach to measure the protein content in different cellular fractions, we utilized a technique called spatial proteomics that measures the cellular distribution of thousands of proteins using a combination of cellular fractionation and MS 13. This method involves first culturing cells with SILAC medium to ensure that proteins are fully labeled 8. The SILAC incorporated cells are then separated into fractions, *e.g*. the cytoplasm (Cyto), nucleus and nucleolus that are recombined such that each fraction has a distinct isotope signature. The labeling thus allows quantification of the relative abundance of peptides originating from subcellular fraction and was used to study the relative distribution of the proteome between the Cyto, nucleus and nucleolus 13. We hypothesized that we could use this technique also to determine the difference in protein localization caused by specific gene knockouts, such as here between wild-type and p53 null cells. We then identified and quantified the change in proteome localization induced by activation of the DNA damage response in either the presence or absence, of p53.

## 2 Materials and methods

### 2.1 Cell culture

The human colon carcinoma cell line HCT116 that are either wild-type (p53^+/+^) or p53 null (p53^−/−^) were cultured as adherent cells in DMEM (Invitrogen, custom order) depleted of arginine and lysine. The DMEM was supplemented with 10% fetal bovine serum dialyzed with a cut-off of 10 kDa (Invitrogen, 26400-044), 100 U/mL penicillin/streptomycin, 2 mM l-glutamine. Arginine and lysine were added in either light (Arg0, Sigma, A5006; Lys0, Sigma, L5501), medium (Arg6, Cambridge Isotope Lab (CIL), CNM-2265; Lys4, CIL, DLM-2640) or heavy (Arg10, CIL, CNLM-539; Lys8, CIL, CNLM-291) form to a final concentration of 28 μg/mL for arginine and 49 μg/mL for lysine. Cells were tested for full incorporation of the label after six passages.

### 2.2 Cell fractionation

Cyto, nuclei (Nuc) and No were prepared from HCT116 cells using a method originally described in 14. Briefly, cells were washed three times with PBS, resuspended in 5 mL buffer A (10 mM HEPES–KOH [pH 7.9], 1.5 mM MgCl_2_, 10 mM KCl, 0.5 mM DTT), and dounce homogenized ten times using a tight pestle. Dounced Nuc were centrifuged at 228×*g* for 5 min at 4°C. The supernatant represents the cytoplasmic fraction. The nuclear pellet was resuspended in 3 mL 0.25 M sucrose, 10 mM MgCl_2,_ and layered over 3 mL 0.35 M sucrose, 0.5 mM MgCl_2_ and centrifuged at 1430×*g* for 5 min at 4°C. The clean, pelleted Nuc were resuspended in 3 mL 0.35 M sucrose, 0.5 mM MgCl_2_ and sonicated for 6×10 s using a microtip probe and a Misonix XL 2020 sonicator at power setting 5. The sonication was checked using phase contrast microscopy, ensuring that there were no intact cells and that the No were readily observed as dense, refractile bodies. The sonicated sample was then layered over 3 mL 0.88 M sucrose, 0.5 mM MgCl_2_ and centrifuged at 2800×*g* for 10 min at 4°C. The pellet contained the No, while the supernatant consisted of the nucleoplasmic fraction. The No were then washed by resuspension in 500 μL of 0.35 M sucrose, 0.5 mM MgCl_2_, followed by centrifugation at 2000×*g* for 2 min at 4°C. Proteins were quantified using the Quant-IT protein assay (Invitrogen) and measured using a Qubit (Invitrogen). Equal amounts of total protein from each fraction were then recombined to recreate a whole-cell extract, but with Cyto, Nuc and No arising from cells with different isotopic labels.

### 2.3 Western blotting and Coomassie staining

Equal amounts (10 μg) of proteins from each fraction were boiled in the loading buffer, and then separated by one-dimensional SDS-PAGE (4–12% Bis-Tris Novex mini-gel, Invitrogen) and either visualized by Coomassie staining (SimplyBlue SafeStain, Invitrogen) or transferred to nitrocellulose prior to western blotting with a rabbit polyclonal RPL11 antibody (abcam) or a mouse monoclonal B23-nucleophosmin antibody (Sigma).

### 2.4 Immunofluorescence

Cells were grown on glass coverslips and fixed with 3.7% paraformaldehyde in cytoskeleton (CSK, 10 mM PIPES pH 6.8, 10 mM NaCl, 300 mM sucrose, 3 mM MgCl_2_ and 2 mM EDTA) buffer for 10 min. Cells were then permeabilized in PBS containing 0.5% Triton X-100 for 10 min, and then labeled with antibodies recognizing RPL11 (Abcam). After washing with PBS containing 0.1% Triton X-100 and PBS, cells were then labeled with a secondary antibody coupled to Alexa 594 (Molecular Probes) and mounted on slides with Vectashield (Vector Laboratories) containing DAPI. Fluorescence imaging was performed on a DeltaVision Spectris widefield deconvolution microscope (Applied Precision), using a CoolMax charge-coupled device camera (Roper Scientific). Cells were imaged using a 60× NA 1.4 Plan-Apochromat objective (Olympus) and the appropriate filter sets (Chroma Technology), with 20 optical sections of 0.5 μM each acquired. SoftWorX software (Applied Precision) was used for both acquisition and deconvolution.

### 2.5 Gel electrophoresis and in-gel digestion

The reconstituted cell fractions were reduced in 10 mM DTT and alkylated in 50 mM iodoacetamide prior to boiling in loading buffer, and then separated by one-dimensional SDS-PAGE (4–12% Bis-Tris Novex mini-gel, Invitrogen) and visualized by colloidal Coomassie staining (Novex, Invitrogen). The entire protein gel lanes were excised and cut into eight slices each. Every gel slice was subjected to in-gel digestion with trypsin 15. The resulting tryptic peptides were extracted by 1% formic acid, then 100% ACN, lyophilized in a speedvac and resuspended in 1% formic acid.

### 2.6 LC-MS/MS

Trypsin-digested peptides were separated using an Ultimate U3000 (Dionex) nanoflow LC-system consisting of a solvent degasser, micro and nanoflow pumps, flow control module, UV detector and a thermostated autosampler. Ten microliter of sample (a total of 2 μg) was loaded with a constant flow of 20 μL/min onto a PepMap C18 trap column (0.3 mm id×5 mm, Dionex). After trap enrichment peptides were eluted onto a PepMap C18 nano column (75 μm×15 cm, Dionex with a linear gradient of 5–35% solvent B (90% ACN with 0.1% formic acid) over 65 min with a constant flow of 300 nL/min. The HPLC system was coupled to an LTQ Orbitrap XL (Thermo Fisher Scientific) *via* a nano ES ion source (Proxeon Biosystems). The spray voltage was set to 1.2 kV and the temperature of the heated capillary was set to 200°C. Full-scan MS survey spectra (*m/z* 335–1800) in profile mode were acquired in the Orbitrap with a resolution of 60 000 after accumulation of 500 000 ions. The five most intense peptide ions from the preview scan in the Orbitrap were fragmented by collision-induced dissociation (normalized collision energy 35%, activation Q 0.250 and activation time 30 ms) in the LTQ after the accumulation of 10 000 ions. Maximal filling times were 1000 ms for the full scans and 150 ms for the MS/MS scans. Precursor ion charge state screening was enabled and all unassigned charge states as well as singly charged species were rejected. The dynamic exclusion list was restricted to a maximum of 500 entries with a maximum retention period of 90 s and a relative mass window of 10 ppm. The lock mass option was enabled for survey scans to improve mass accuracy 16. Data were acquired using the Xcalibur software.

### 2.7 Quantification and bioinformatic analysis

Quantitation was performed using the program MaxQuant version 1.0.13.13 17, 18. The derived peak list generated by Quant.exe (the first part of MaxQuant) was searched using MASCOT (Matrix Sciences, London, UK) as the database search engine for peptide identifications against the International Protein Index (IPI) human protein database version 3.37 containing 69 290 proteins, to which 175 commonly observed contaminants and all the reversed sequences had been added. The initial mass tolerance was set to 7 ppm. and MS/MS mass tolerance was 0.5 Da. Enzyme was set to trypsin/p with two missed cleavages. Carbamidomethylation of cysteine was searched as a fixed modification, whereas *N*-acetyl protein and oxidation of methionine were searched as variable modifications. Identification was set to a false discovery rate (FDR) of 1%. To achieve reliable identifications, all proteins were accepted based on the criteria that the number of forward hits in the database was at least 100-fold higher than the number of reverse database hits, thus resulting in a FDR of less than 1%. A minimum of two peptides were quantified for each protein. Protein isoforms and proteins that cannot be distinguished based on the peptides identified are grouped and displayed on a single line with multiple International Protein Index numbers (see Supporting Information tables).

## 3 Results

**Figure 1:**
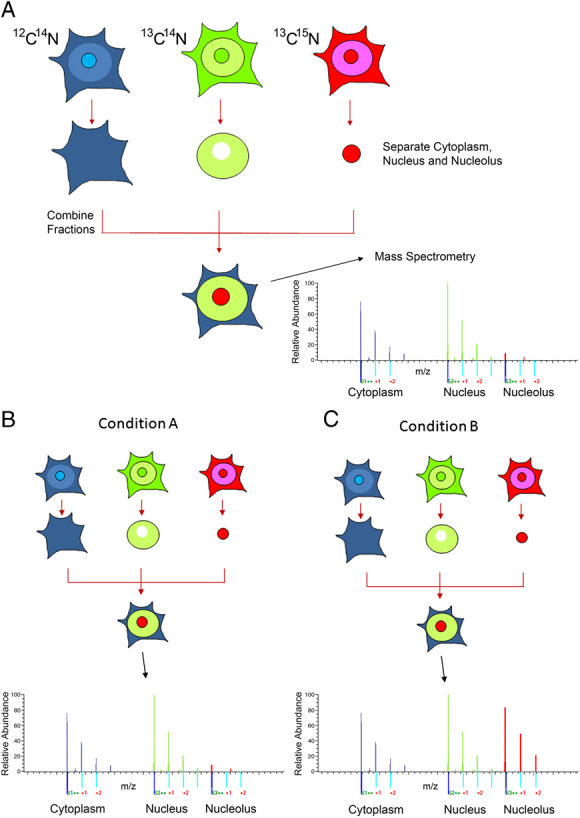
Spatial Proteomics Method (A) Human colon carcinoma HCT116 cells were grown in DMEM containing either the normal “light” isotopes of carbon and nitrogen (*i.e*. ^12^C^14^N) (light) or l-arginine-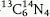 and l-lysine-^2^H_4_ (medium) or l-arginine- ^13^C_6_-^15^N_4_ and l-lysine-^13^C_6_-^15^N_2_ (heavy). Separate cytoplasmic, nuclear and nucleolar fractions were isolated from each labeled cell population. Equal amounts of total protein from each fraction were then recombined to recreate a whole cell extract, but with Cyto, Nuc and No arising from cells with different isotope labels. The recombined whole cell extract mixture was solubilized with loading buffer, proteins separated using SDS-PAGE and the resulting gel cut into eight equal pieces, trypsin digested and analyzed by LC-MS/MS using an LTQ Orbitrap. To compare different genotype or conditions, cells are analyzed as described above, and changes in localization under different conditions will result in changes in SILAC ratios under normal conditions (A) and following treatment or when studying a different condition (C).

HCT116 p53^+/+^ is a human colon carcinoma cell line, which contains a wild-type p53 gene, whereas the HCT116 p53^−/−^ is a p53 knockout cell line derived from HCT116 p53^+/+^ by homologous recombination 19. Cells were grown in three different media for SILAC labeling, containing arginine and lysine, either with the normal “light” isotopes of carbon and nitrogen (*i.e*. ^12^C^14^N) (light), or l-arginine-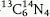 and l-lysine-^2^H_4_ (medium) or l-arginine- ^13^C_6_-^15^N_4_ and l-lysine-^13^C_6_-^15^N_2_ (heavy). Separate Cyto, Nuc and No fractions were isolated from each labeled cell population as previously described 14. Equal amounts of total protein from each fraction were then recombined to recreate a whole cell extract, but with Cyto, Nuc and No arising from cells with different isotope labels (Fig. 1A). The recombined whole cell extract mixture was solubilised with the loading buffer, proteins separated using SDS-PAGE and the resulting gel cut into eight equal pieces, trypsin digested and analyzed by LC-MS/MS using an LTQ Orbitrap 20. The resulting ratios between light, medium and heavy isotopic forms for each peptide identified were quantified using MaxQuant 17. The separate ratio values for each peptide in a given protein were averaged to provide a measure of the relative distribution for the protein between the respective Cyto, Nuc and No compartments (Fig. 1A). Two independent experiments were performed of the whole spatial proteomics procedure using separate preparations of isotope-labeled HCT116 cells (Figs. 1B and C). We named each repeat WTa and WTb for the wild-type cells, and p53a and p53b for the p53 knock-out cells. A total of 20 500 peptides were quantified, corresponding to 1958 proteins (*i.e*. protein isoform groups, see Section 2) using a minimum of two peptides identified and a FDR of 1%. The distribution ratio between the three cellular compartments derived for each protein was calculated as a median of the values for all peptides identified for each protein (Supporting Information Table 1).

**Figure 2:**
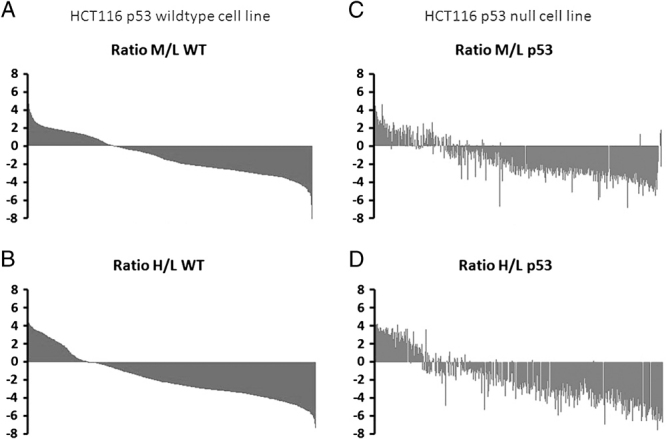
Comparison of HCT116 p53^+/+^ and p53^−/−^ cells. Graph represents the proteins (*x* axis) *versus* the log base two of the SILAC ratio corresponding to the (A), (C) nucleoplasmic/cytoplasmic (medium over light, M/L) or the (B), (D) nucleolar/cytoplasmic (heavy over light, H/L) in the *y* axis. The plots correspond to the cellular distribution of proteins from WT cells (A and B) or from the p53 knock-out cells (C and D). The order of the proteins shown is sorted according to their localization ratio from the WT cells, and the proteins are displayed in the same order in the p53 knock-out cells.

To check whether any differences observed in proteome localization dependent upon p53 arose because of biologically relevant changes and not simply due to experimental variation (*e.g*. in reproducibility of either MS or fractionation procedures), statistical evaluation of the repeat data sets was carried out. The data show a Pearson correlation of 0.92 and 0.93 for the nucleoplasmic/cytoplasmic and the nucleolar/cytoplasmic ratios, respectively between repeats of the wild-type cells (*i.e*. WTa *versus* WTb). A similar reproducibility was observed for the p53 knock-out cells, with Pearson correlations of 0.93 and 0.98 for the nucleoplasmic/cytoplasmic and the nucleolar/cytoplasmic ratios, respectively, between repeats using the p53 knock-out cells. Interestingly, there is little variation between the wild-type and the p53 knock-out cells in terms of the overall proteome localization within the cell. The Pearson correlation between the p53^+/+^ and ^−/−^ cells is 0.91 and 0.94 for the nucleoplasmic/cytoplasmic and the nucleolar/cytoplasmic ratios, respectively, indicating there are little or no p53-dependent differences in subcellular protein localization. This is illustrated in the respective plots showing comparison of the subcellular protein distribution of WT cells according to their nucleoplasmic/cytoplasmic ratio (Fig. 2A) and nucleolar/cytoplasmic ratio (Fig. 2B) and of the p53 knock-out cells according to their nucleoplasmic/cytoplasmic ratio (Fig. 2C) and nucleolar/cytoplasmic ratio (Fig. 2D). The proteins were sorted according to their log base two WT ratios from highest to lowest, and the proteins are displayed in the same order in the p53 knock-out cells to underline the differences. Although there are minor variations between the two cell lines with regards to proteome localization, only 40 proteins had a ratio difference greater than two (Supporting Information Table 2). Interestingly, 18 of those 40 proteins are involved directly in pre-mRNA splicing, which could suggest that it reflects a genuine difference between the two cell genotypes. This difference in proteins involved in mRNA processing has also been observed previously in a p53-dependent protein expression profiling study of the HCT116 cells 21. We conclude that variations from the fractionation and MS procedure have at most a small effect on the values measured and we conclude further that there are only minor differences in subcellular protein localization resulting directly from p53 genotype.

**Figure 3:**
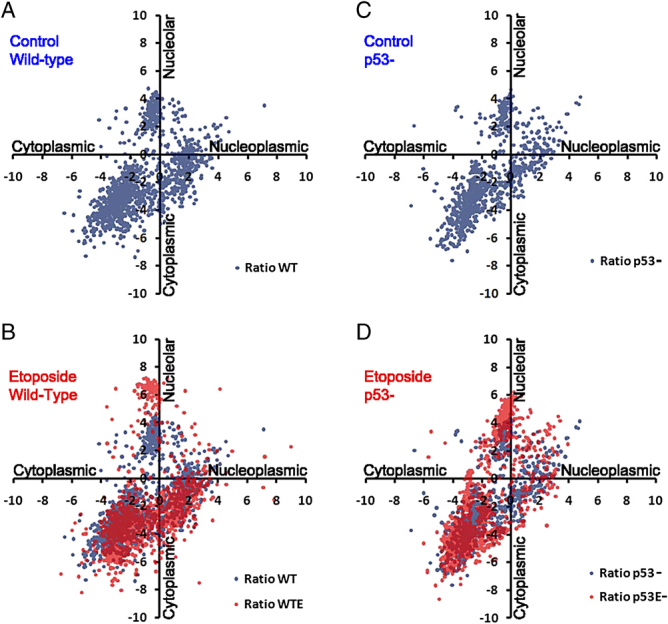
DNA damage response and p53. Visualization of the spatial proteomics data by graphical representation, plotting the log base two Nucleoplasmic/Cytoplasmic ratio on the *x* axis and log base two Nucleolar/Cytoplasmic ratio on the *y* axis of either mock treated (A) wild-type and (B) p53 knock-out cells or of Eto-treated (C) wild-type and (D) p53 knock-out cells.

Next, we used spatial proteomics to compare the cellular localization of the proteome in HCT116 cells following exposure to the topoisomerase II inhibitor etoposide (Eto), which induces DNA damage and double strand breaks. We checked whether we could observe any differences in subcellular localization following DNA damage that would be dependent on p53. The data show a moderate effect on protein localization in wild-type cells with Pearson correlations of 0.88 and 0.85 for the nucleoplasmic/cytoplasmic and the nucleolar/cytoplasmic ratios, respectively, between samples when wild-type cells were treated with Eto. This is illustrated in the respective scatter plots showing the localization of proteins of wild-type cells (Fig. 3A, blue) and showing the effect of Eto on protein localization (Fig. 3B, red). For the p53 knock-out cells (Fig. 3C, blue), we found a similar effect of Eto treatment, with a Pearson correlation of 0.89 for the nucleoplasmic/cytoplasmic ratio (Fig. 3D, red). However, the effect on the nucleolar localization was much closer to the differences between repeats of the experiment with a Pearson correlation of 0.93 for the nucleolar/cytoplasmic ratios between samples when cells were treated with Eto. This suggests that Eto induces a significant change in the protein content of the nucleolus, and that this change is at least in part dependent on the presence of wild-type p53. This increased segregation of nucleolar proteins within the nucleolus following stress is consistent with the previous observations 5.

**Figure 4:**
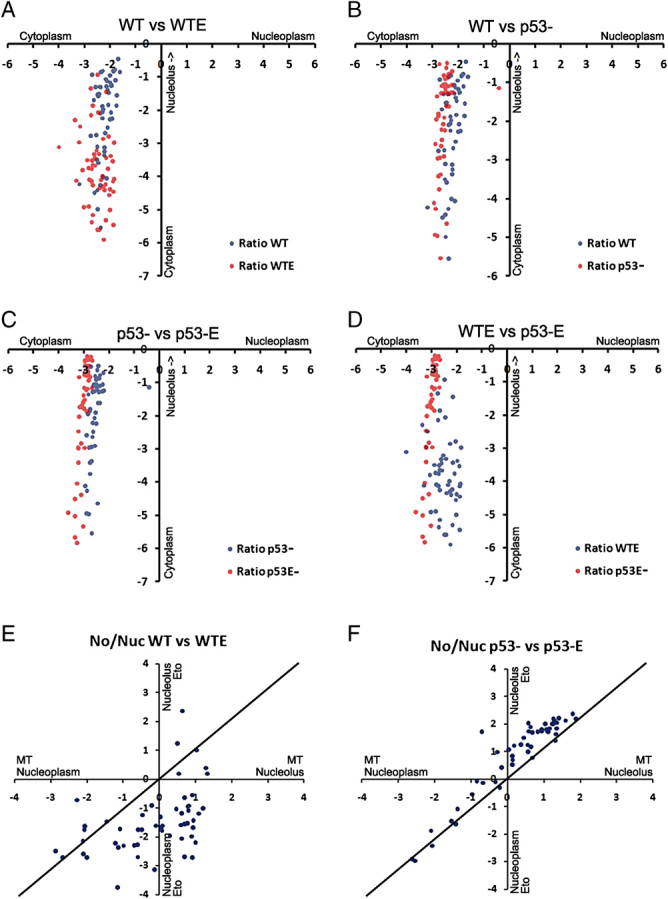
Effect of p53 on the ribosomal proteins response to DNA damage. Visualization of the spatial proteomics data of ribosomal proteins by graphical representation, plotting the log base two Nucleoplasmic/Cytoplasmic ratio on the *x* axis and log base two Nucleolar/Cytoplasmic ratio on the *y* axis of (A) wild-type mock treated (blue) and wild-type Eto-treated cells (red), (B) wild-type mock treated (blue) and p53 knock-out mock treated cells (red), (C) p53 knock-out mock treated (blue) and p53 knock-out Eto-treated cells (red) and (D) wild-type Eto treated (blue) and p53 knock-out Eto-treated cells (red). Visualization of the spatial proteomics data of ribosomal proteins by graphical representation, plotting the log base two Nucleolar/Nucleoplasmic ratio of mock-treated cells on the *x* axis and of Eto-treated cells on the *y* axis for the (E) wild-type cells and (F) p53 knock-out cells.

These spatial proteomics data provide new evidence showing that p53 is involved in the response to DNA damage in the nucleolus. We analyzed further which proteins specifically showed a difference in localization in p53 null cells. We found 123 proteins that after DNA damage had more than a twofold difference between the wild-type and the p53 knock-out cells (Supporting Information Table 3, p.1). The most striking group of proteins identified was the ribosomal proteins, which show a clear change in cellular localization following DNA damage in wild-type cells (Fig. 4A and Supporting Information Table 3, p.2). These data are consistent with an inhibition of the nucleolar import of ribosomal proteins. Interestingly, this effect is not observed in the absence of p53 (Fig. 4C), indicating that p53 is directly or indirectly involved in the inhibition of ribosomal protein import following cellular stress (Fig. 4D). There was no difference between the p53 wild-type and null cells in the localization of ribosomal proteins in the absence of DNA damage (Fig. 4B). We also observed a difference between the nucleoplasm and the nucleolus. If simultaneously there is an equivalent change in cytoplasmic and nucleoplasmic content from the nucleolus, it is not possible to visualize this change by plotting the nucleoplasmic/cytoplasmic *versus* the nucleolar/cytoplasmic ratios. Thus, we plotted the nucleolar/nucleoplasmic ratios before and after DNA damage (Figs. 4E and F). We observed a redistribution of the ribosomal proteins following DNA damage in the wild-type cells from the nucleolus to the nucleoplasm (Fig. 4E). In this case, most proteins appear below the line line through the origin on the scatter plot. However, this relocalization did not occur in the absence of p53 (Fig. 4F) where most proteins cluster near the line through the origin.

**Figure 5:**
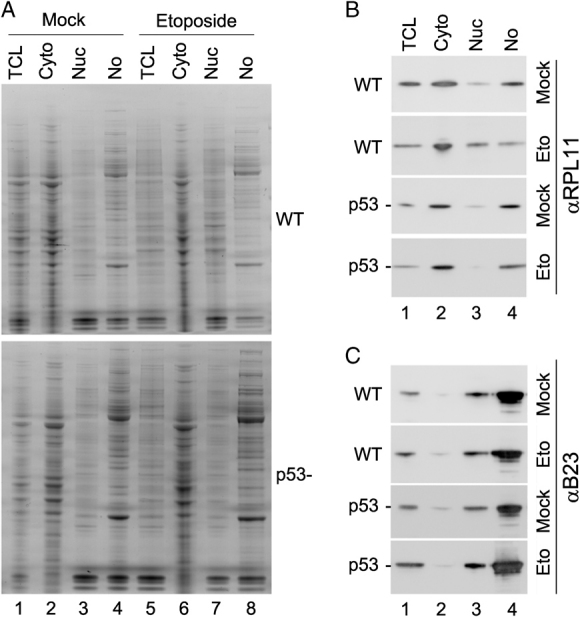
Western blot analysis of ribosomal proteom response. (A) Equal amount (10 μg) of total cell lysates (lanes 1 and 5) or of extracts from the cytoplasmic (Cyto, lanes 2 and 6), nuclear (Nuc, lanes 3 and 7) or nucleolar (No, lanes 4 and 8) fractions of HCT116 wild-type cells (top) or p53 knock-out (bottom) that were either mock-treated (1–4) or treated with 50 μM Eto for 1 h (5–8) were separated by SDS-PAGE and stained with Coomassie Blue. The same fractions were separated by SDS-PAGE, transferred to nitrocellulose prior to Western blotting with an RPL11 antibody (B) or a B23-nucleophosmin antibody (C).

**Figure 6:**
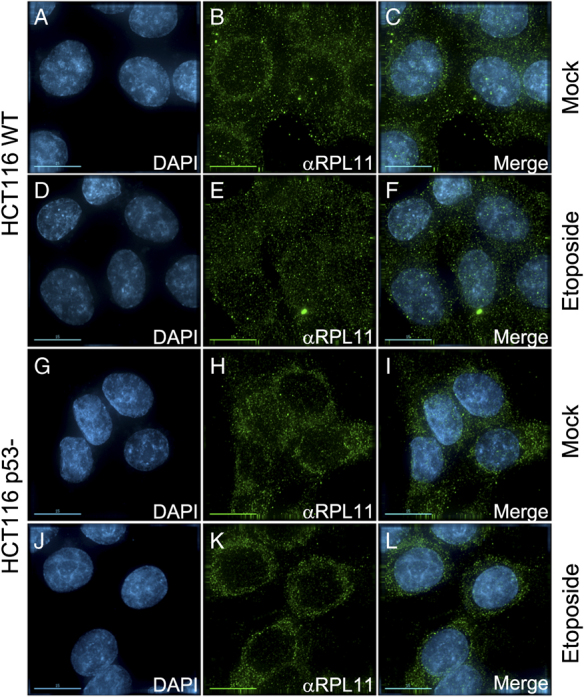
Immunofluorescence analysis of ribosomal protein response. HCT116 wild-type (A–F) and p53 knock-out (G–L) were cultured on coverslips and either mock-treated (A–C and G–I) or treated with 50 μM Eto (D–F and J–L) for 1 h. Cells were then fixed with 3.7% paraformaldehyde in CSK buffer for 10 min, permeabilized and labeled for immunofluorescence using an antibody recognizing RPL11 (B–E–H–K). DNA was visualized using DAPI (A–D–G–J). Scale bars represent 15 μm.

To confirm the change in localization of ribosomal proteins after DNA damage, we performed both western blots on cellular fractions and immunofluorescence. Equal amounts of either total cell lysates (Fig. 5A, lanes 1 and 5) or of extracts from the cytoplasmic (Fig. 5A, Cyto, lanes 2 and 6), nuclear (Fig. 5A, Nuc, lanes 3 and 7) or nucleolar (Fig. 5A, No, lanes 4 and 8) fractions of either HCT116 wild-type cells (top) or p53 knock-out (bottom) that were either mock-treated (lanes 1–4) or treated with 50 μM Eto (lanes 5–8) for 1 h were separated by SDS-PAGE and stained with Coomassie Blue to make sure of equivalent loading of proteins from each fraction. The same fractions were again separated by SDS-PAGE, but this time transferred to nitrocellulose prior to western blotting with either an RPL11 antibody or a B23-nucleophosmin antibody. RPL11 is observed in both the Cyto and nucleolus in the absence of DNA damage (Fig. 5B, mock). However, there is a reduction in the amount of RPL11 located in the nucleolus following treatment with Eto (Fig. 5B, lane 4). This appears to coincide with an increase in RPL11 in both the Cyto and the nucleoplasm, and this increase is not observed in p53 knock-out cells (Fig. 5B, Eto, lanes 3 and 4). To further demonstrate this relocalization, HCT116 wild-type (Figs. 6A–F) and p53 knock-out (Figs. 6G–L) cells were cultured on coverslips and either mock-treated (Figs. 6A–C and Fig. 6G–I) or treated with 50 μM Eto (Figs. 6D–F and J–L) for 1 h. Cells were then fixed, permeabilized and labeled for immunofluorescence using an antibody recognizing RPL11 (Figs. 6B–E and H–K). We did not detect endogenous RPL11 in the nucleolus in these experiments despite trying different fixation procedures, although we did detect a GFP-tagged ribosomal protein in both the Cyto and the No 22. This is likely due to failure of the antibody to penetrate and reach its epitope in the nucleolus of fixed cells. However, we detected a strong cytoplasmic signal by immunofluorescence with the RPL11 antibody. There was a difference in the localization of RPL11 following treatment of HCT116 cells with Eto (compare Figs. 6B and E), with an increase in labelling in the nucleus evident after DNA damage. Again, this relocalization of RPL11 was not observed in HCT116 cells lacking p53 (compare Figs. 6H and K). This demonstrates how spatial proteomics can be used to not only look at individual protein responses under different conditions, but also how different groups of proteins can be analyzed provide an unbiaised understanding of underlying functional interactions at a system-wide level.

**Figure 7:**
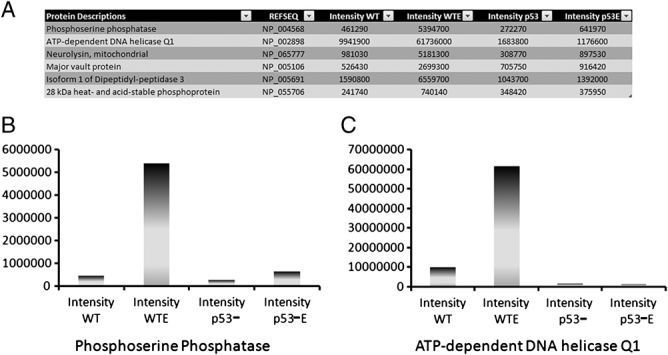
p53-dependent protein induced by DNA damage. Example of two proteins (Phosphoserine Phosphatase and ATP-dependent DNA helicase Q1) whose intensity was increased following Eto treatment in wild-type cells (compare intensity WT *versus* intensity WTE), but not in p53 knock-out cells (compare intensity p53 *versus* intensity p53E).

The proteomic experiments not only provide data showing the relative enrichment of proteins in different subcellular compartments, but they also make it possible to analyze the ion intensity of proteins in the cell as a measurement of total protein abundance. Although less reliable than the SILAC ratios, it still provides an interesting measure of changes in protein level. We thus analyzed the intensity values to address whether we could identify candidate proteins induced following treatment with Eto, and which of these changes in protein level are dependent on p53. Approximately 5% (103) of proteins identified showed a >three-fold increase in protein level following treatment with Eto (Supporting Information Table 4, p.1). Of those 103 proteins, we found a specific GO enrichment in proteins involved in mRNA splicing (12), amino acid metabolic process (10), intracellular transport (10) and response to oxidative stress (6). Interestingly, only six of these proteins were not increased also in the p53 knock-out cells (Fig. 7A and Supporting Information Table 4, p.2). The increase in intensity of Phosphoserine phosphatase and the ATP-dependent DNA helicase Q1 (RecQ1) are shown in Figs. 7B and C, where the induction of protein after DNA damage is seen only in wild-type cells. None of those six proteins have been previously characterized as being p53-regulated transcriptional targets, although a disctinct role for RecQ1 has been described in the maintenance of the genomic stability. RecQ1 catalyzes DNA unwinding and strand annealing, and these activities are likely to be important for its role in DNA repair 23.

## 4 Discussion

In this study we used spatial proteomics as an unbiased and quantitative MS-based approach to study not only the difference in localization between cells that differ only at one gene locus, p53, but also to see the difference in how the cells respond to DNA damage, a response in which p53 is involved. We show that there are very few differences in the overall subcellular localization of proteins that depends on the p53 gene. This confirms the specificity of the response and is consistent with the fact that under normal conditions, p53 is a short-lived protein that is present in cells at a barely detectable level. Thus, p53 makes little difference to the subcellular proteome localization under normal growth conditions. Interestingly, the 40 proteins that showed a localization difference in the absence of p53 (but without DNA damage) were mostly proteins involved in splicing, including most of the members of the core spliceosome (Sm A, B, D1, D3 and E), as well as several other splicing factors (SF3A, B, hnRNPs) This may reflect a true difference between those two cell lines in p53-dependent gene expression and merit further investigation in future. However, we cannot rule out that the difference in expression was acquired through secondary mutations as a result of culturing the two cell lines in parallel for several years 1.

Upon exposure of cells to various forms of exogenous stress, such as DNA damage, there is a stabilization of p53, which is then responsible for an ensuing cascade of events, resulting in either cell cycle arrest or in apoptosis. We observed a general effect of Eto on the nucleoplasmic/cytoplasmic protein ratios, which shows that the response in both cell lines is generally quite similar for the proteins shuttling between the Cyto and the nucleus. We found a notable difference in the nucleolar/cytoplasmic ratios following DNA damage, whether p53 is present or not. In wild-type cells, p53 appears to cause a shut-down of nucleolar activity, which results in a specific segregation of nucleolar proteins within the nucleolus. However, this seems to be dependent on p53, as the effect is observed to a much lesser extent in p53 knock-out cells. One such consequence is that the ribosomal proteins are no longer accumulating in the nucleolus following DNA damage. This suggest a possible early role for p53 in shutting down the rDNA transcription machinery, as well as either stopping the nucleolar recruitment, or retention of ribosomal proteins in the nucleolus, indicating that cells undergo a rapid stop in ribosome subunit production following DNA damage. Several recent reports showed that p53 becomes activated after silencing of ribosomal proteins such as RPL23 24, RPL11 25, RPS6 26 and TIF1A 27. Other evidence emerging from a number of mouse models support the existence of this ribosomal dependent p53 checkpoint *in vivo* 28. During normal cellular growth, ribosomal proteins are assembled into ribosomal subunits, but several ribosomal proteins (RPL11, RPL5, RPL23, RPS7 and RPS9) have now been shown to be released from the nucleolus following stress and to bind HDM2, resulting in stabilization of p53 28–31. Our data suggest that p53 is actually necessary for the initial release of ribosomal proteins from the nucleolus following stress, and that this release probably results in an amplification of the p53 response through stabilization by preventing HDM2-mediated degradation of p53.

Cell growth is essential for cell cycle progression and the attainment of a particular cell mass is a prerequisite for cell division. As ribosome levels reflect the capacity of the cell to grow and achieve this cell mass, it is possible that ribosome subunit production in the nucleolus plays an important role in controlling cell cycle progression. Another important link between the fate of the cell and the level of ribosome subunit biogenesis has been demonstrated from the observation that events that perturb the structure or function of the nucleolus, the center of rDNA transcription and ribosome subunit production, can cause p53 accumulation 5, 32, which can lead to cell cycle arrest or apoptosis 1. Because such events produce a negative effect on rRNA transcription, it is possible that the levels of rRNA are directly influenced by p53 accumulation and the decision to trigger cell cycle arrest, or even apoptosis 33. In addition, the repetitive nature of the rRNA genes, in combination with the dense loading of the RNA polymerase I complexes on the active templates, provides a potential sensor for DNA damage. The amplification of signals associated with stalled polymerases and/or reduced rRNA levels could further activate p53 and other DNA-damage response pathways.

The spatial proteomics method used here allowed us to characterize how cells respond differently depending on the presence of a single, albeit very important, protein in response to DNA damage. It has provided accurate information at a proteome-wide level of the specific effect, and has allowed us to characterize more precisely how cells respond to exposure to Eto. It will be interesting in the future to analyze also the post-translational modifications that change under different conditions. The spatial proteomics approach complements other methods and can be used to further analyze and independently verify data from microscopy and molecular studies that are not readily applicable in high throughput. We envisage that spatial proteomics can be used to characterize a wide range of different cell types and can be combined with alternative fractionation techniques to analyze multiple subcellular compartments and structures.
