# Supplementary material for: p53-Dependent subcellular proteome localization following DNA damage
Source: Proteomics. 2010 Nov;10(22):4087–97. doi: 10.1002/pmic.201000213 (PMC3229981; doi:10.1002/pmic.201000213)
Supplement: Supplementary file 5 [file pmic0010-4087-SD5.pdf]

PROTEOMICS

Supporting Information for Proteomics

DOI 10.1002/pmic.201000213

François-Michel Boisvert and Angus I. Lamond

p53-Dependent subcellular proteome localization following DNA damage

© 2010 WILEY-VCH Verlag GmbH & Co. KGaA, Weinheim
